# Improving the Performance of an EEG-Based Motor Imagery Brain Computer Interface Using Task Evoked Changes in Pupil Diameter

**DOI:** 10.1371/journal.pone.0121262

**Published:** 2015-03-27

**Authors:** David Rozado, Andreas Duenser, Ben Howell

**Affiliations:** 1 CSIRO—Digital Productivity Flagship. 15 College Rd, Sandy Bay, TAS 7005, Australia; Duke University, UNITED STATES

## Abstract

For individuals with high degrees of motor disability or locked-in syndrome, it is impractical or impossible to use mechanical switches to interact with electronic devices. Brain computer interfaces (BCIs) can use motor imagery to detect interaction intention from users but lack the accuracy of mechanical switches. Hence, there exists a strong need to improve the accuracy of EEG-based motor imagery BCIs attempting to implement an on/off switch. Here, we investigate how monitoring the pupil diameter of a person as a psycho-physiological parameter in addition to traditional EEG channels can improve the classification accuracy of a switch-like BCI. We have recently noticed in our lab (work not yet published) how motor imagery is associated with increases in pupil diameter when compared to a control rest condition. The pupil diameter parameter is easily accessible through video oculography since most gaze tracking systems report pupil diameter invariant to head position. We performed a user study with 30 participants using a typical EEG based motor imagery BCI. We used common spatial patterns to separate motor imagery, signaling movement intention, from a rest control condition. By monitoring the pupil diameter of the user and using this parameter as an additional feature, we show that the performance of the classifier trying to discriminate motor imagery from a control condition improves over the traditional approach using just EEG derived features. Given the limitations of EEG to construct highly robust and reliable BCIs, we postulate that multi-modal approaches, such as the one presented here that monitor several psycho-physiological parameters, can be a successful strategy in making BCIs more accurate and less vulnerable to constraints such as requirements for long training sessions or high signal to noise ratio of electrode channels.

## Introduction

Individuals in advance stages of motor neuron disease, those suffering specific types of brain stem strokes that lead to locked-in syndrome, or those with other severe motor disability, are unable to generate voluntary muscle movements. Since sensory and cognitive functions are often spared in such patients, they are in desperate need of innovative communication channels with electronic devices. Brain computer interfaces (BCI) are in principle capable of establishing a direct connection between the human brain and electronic or mechanical devices. One of the simplest BCIs that can be conceptualized is the idea of a mental switch. A mental switch refers to a BCI system through which a subject can generate simple binary switch commands without the involvement of motor output effectors. Several types of neuro-imaging tools have been traditionally used to build functional BCIs: electroencephalography (EEG) [1], functional magnetic resonance imaging (fMRI) [2], magneto-encephalography (MEG) [3], functional near infrared spectroscopy (fNIR) [4], electro-corticography [5] and intra-cortical recordings [6]. All of these neuro-imaging techniques have been shown in the literature capable of generating a BCI based mental switch. However, the accuracy, robustness and latency of such systems is sub-optimal when compared to traditional physical switches. Hence, a robust, accurate and fast BCI based switch interface remains elusive. In this work, we build upon previous findings of pupil dilation during mental imagery [7] in order to improve the performance of an EEG-based mental switch. We do this by using the pupil diameter of subjects engaged in motor imagery as an additional feature to a linear classifier on top of the EEG-derived features employed by traditional BCI classifiers. The pupil diameter time series is captured using a remote video-based eye tracker.

Motor imagery BCIs often try to distinguish imaginary motion of different limbs (i.e. right versus left hand or hand versus feet). We focus here instead in using motor imagery of a limb (left hand) as a switch. Such BCI switch is triggered by engaging in imaginary left hand grasping motion. The BCI switch remains silent in a rest condition in which the subject does not imagine hand motion.

To sum up, we intend to demonstrate that the enlargement of the pupil triggered during execution of imaginary hand movements can be detected by video oculography and that this feature can be used to improve the accuracy of a traditional EEG-based motor imagery BCI. Hence, we have explored how to improve the detection of motor imagery by adding a pupil diameter feature to the set of features extracted from the EEG signal during a rather short (5 minutes) training session with two classes of trials: left motor imaginary and a no task (or rest) condition. Our contribution shows that this multi-modal paradigm leverages the pupil diameter feature to improve the accuracy of motor imagery detection as compared to a traditional BCI using only EEG signal derived features.

## Related Work

### Brain Computer Interfaces

Despite the limitations of the EEG signal for the construction of reliable BCIs: susceptibility to noise, pervasiveness of electro-magnetic and muscle artifacts, limited spatial accuracy, inconvenience of gel-based electrode attachment to subject’s scalps and low information rate throughput [8], EEG-based BCIs are the predominant form of BCIs in the scientific literature due to the low cost of the technology, non-invasiveness, high temporal resolution and mobility that the technology affords [9].

BCIs can be used to detect the degree of relaxation of a subject [10], detect incurred errors [11], type on an onscreen keyboard using the P-300 paradigms [12] or to determine specific oscillatory stimuli to which the subject is paying attention to by the detection of steady-state evoked potentials [13]. A sub-type of BCIs uses a specific type of electrical behavior produced by populations of neurons over the motor-sensory cortex when the subject engages in motor-imagery [14]. Motor imagery refers to a mental rehearsal of motor activity without any overt motor output. It has been shown before that mental imagery engages the same brain regions that are involved in preparing and executing motor movements [15]. The sensory motor cortex generates two types of oscillatory activity that can be used for a BCI based on motor imagery: the Rolandic mu rhythm (7–13 Hz) and the Central beta rhythm (15–30 Hz). Motor behavior and mental motor imagery results in event related desynchronisation (ERD) of mu rhythms [16].

Using motor imagery as a trigger for a given action has been previously studied in the field of electroencephalography (EEG), with moderate success: overall accuracy in state-of-the-art systems is currently well above chance [17, 18], but under very controlled conditions such as maintaining gaze fixed on a screen location, detecting motor imagery within a constrained time window determined by the computer (synchronous paradigms) and/or with the subject trying to minimize all sort of muscle movements that could interfere with the EEG signal: ocular movements, facial and neck muscles activity. Such BCIs often require relatively long training periods to calibrate a subject-specific model and manual curation of artifacts in the training data.

As a baseline algorithm for EEG-based motor imaginary classification, the method of Common Spatial Patterns (CSP) is currently the state of the art for EEG-based BCI’s specifically designed to discriminate motor imaginary signal classes from each other [15]. The CSP method is based on a decomposition of the raw EEG signal into spatial patterns extracted from raw classes of single trials where these patterns maximize the difference between the two classes. The features extracted through the CSP procedure are then fed to a classifier that tries to find the optimal separation between the classes. Several classification approaches have been tried in the literature such as auto-regressive neuronal networks [19], support vector machines [20] or hidden Markov models [21]. Among them, a simple linear discriminant analysis (LDA), offers a reasonable trade-off between accuracy and speed of computation [22].

### Pupillometry

Pupillometry or the monitoring of pupil diameter can be used to detect a wide range of cognitive activities. One of the main limitations of pupillometry is that any changes in pupil diameter as a result of cognitive processes are 2^*nd*^ order effects (ranging from 0.1 mm to 0.5 mm) in contrast to 1^*st*^ order effects resulting from changes in ambient illumination, stimulus brightness or chemical substances that affect pupil size (ranging from 0.1 mm to 8 mm) [23]. Hence, in order to block confounding variables in cognitive experiments involving pupillometry as a dependent variable, light conditions and stimulus brightness have to be controlled during the experiment.

Pupil dilations can provide a wealth of information regarding an individual’s cognitive processes [24–26]. Pupillometry has been successfully used to detect subject’s engagement in mental arithmetic [27] or working memory load [28, 29] and sexual arousal [30]. Non-motor-specific mental imagery has also been shown to elicit changes in pupil dilation [7]. We have recently noticed in our lab that hand motor imagery also produces an enlargement of the pupil. We started this work with the hypothesis that the pupil diameter feature can aid to better distinguish hand motor imaginary from a rest condition in a BCI context. Hence, The goal of this paper is to show how the addition of the pupil diameter feature can aid traditional CSP-reliant BCIs to reduce the error rate of an LDA classifier trying to discriminate motor imagery from a rest condition. Through a comprehensive user study we are in fact able to show that the addition of the pupil diameter feature can improve classification accuracy.

## Materials and Methods

### Ethics statement

We did not anticipate any foreseeable risks related to the experimental activities and the user study was approved by the institutional review board for human research of the Commonwealth Scientific and Industrial Research Organization (CSIRO). Consent to participate in the experiments was given in writing. Participants were recruited from a pool of volunteers on-site. This article does not include identifying or potentially identifying information about the subjects.

### Hardware

A Tobii X2-30 eye tracker was used in the experimental part to monitor subjects gaze and pupil diameter. Gaze samples were sampled at 30 frames/s. Gaze estimation accuracy was around 0.5 degrees of visual angle at 60 cm from the screen.

A single BioSemi ActiveTwo electroencephalography (EEG) amplifier with 32 channels was used to monitor scalp potentials. Streaming data was sampled at 512Hz.

### Software

We used the open source software package EEGLab [31] for data analysis. BCILAB [32] was used for the detection of motor imagery with the common spatial pattern paradigm used as a baseline against which to test how the addition of the pupil diameter feature improves the performance of motor imagery detection. Lab Streaming Layer (LSL) was used for networking, data collation and time synchronization for EEG, gaze and pupil size time series streams as well as data streams containing stimulus events. LSL contains a number a native application to interface with the Biosemi amplifier and also provides a public API for input and output of data so that developers can add their own modules. The Simulation and Neuroscience Application Platform (SNAP) was used as a stimulus presentation environment. SNAP offers a high level API for presentation of simple graphics and audio with precise timing of stimuli presentation and organization of stimulus events into larger trial blocks allowing for multi-presentation trials. The SNAP module was adapted to stream data (stimulus events, annotations and markers) to the LSL engine. We ran EEGLAB and BCILAB with Matlab vR2014a in a Windows environment.

As an interface module to the Tobii X2-30 eye tracker, which we used to monitor pupil size and gaze position, we developed a custom module to interface with its API using the Python scripting language.

**Fig 1:**
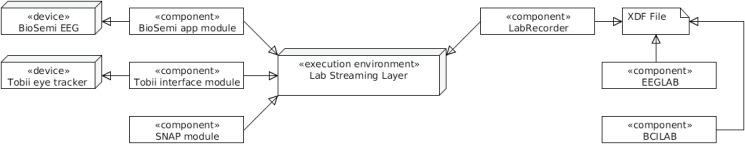
Experiment Software Architecture. The figure illustrates a conceptual overview of the software architecture used in the experiments. The BioSemi module provided a data stream containing the EEG data from the participant. The Tobii X2-30 module provided a data stream containing gaze and pupil diameter data of the participant. The SNAP module with custom scripts provided a data stream containing annotated stimulus events and markers. The Lab Streaming Layer collected and time synchronised all experimental data streams. The LabRecorder module recorded all data to file using the XDF format. Data analysis on the user study generated XDF files was carried out using the BCILAB and EEGLAB software libraries.

The LabRecorder application module is provided with Lab Streaming Layer. This application provides a list containing all current data streams recognized within LSL. We recorded all data streams to a file in eXtensible Data Format (XDF). We used this for storage of the concurrent data streams, per stream meta-data, sampling meta-data and time synchronization meta-data generated in the experiments. The experiment pipeline software architecture is illustrated in Fig. 1.

### Experiment

#### Procedure

The experimental paradigm was designed to discriminate between two mental states: imaginary left hand grasping motions and a no task control condition in which the subject was asked to not engage in motor imagery. The specific left hand motor imaginary instead of right hand motor imagery was chosen for no particular reason but it remained the same for all participants and experimental blocks to maintain consistency across the entire experiment. The experiment was divided in three blocks of five minutes each. One block of five minutes was used for the subject to familiarize himself or herself with the experimental setup. The remaining two blocks were used for actual model training and/or classification.

Subjects were seated in a chair and they were asked to fixate on a cross centered on a computer monitor, at a distance of 60 cm, for the length of each block. Subjects were asked to keep their arms and hands relaxed and to avoid eye movements during the recordings. The gaze tracker used in this work provides pupil estimates invariant to gaze position. However, pupil size is affected by the brightness of the stimuli being gaze at. Since gazing at different areas of the screen would project in the retina visual information with different brightness values according to the different objects around the screen edges, we tried to control for this potential cofounding factor. Hence, we asked participants to try to maintain their gaze fixed in the center cross. To ensure that gaze remained fixated on the central cross, an alarm was engaged which emitted a beep if the participant fixated away from the central cross using the point of regard data provided by the gaze tracker. While we did not specifically control for ambient illumination, ambient light remained relatively stable across the entire duration of the experiment, as the room where experiments were conducted used artificial (standard fluorescent) illumination. Subjects received no feedback in terms of how they were doing within each trial.

**Fig 2:**
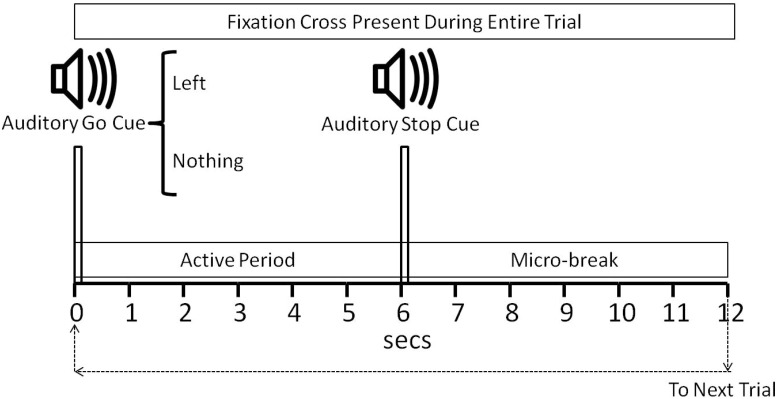
Timing of the Experimental Task Within One Trial. The auditory go cue consisted of the utterance of the words “Left” or “Nothing” according to the class that the trial belongs to. The auditory stop cue consisted of the utterance of the word “Stop”. A micro-break of 6 seconds was given to participants within each trial.

Each experimental block consisted of 25 trials which randomly consisted of the “left” and “nothing” conditions. Each block lasted 5 minutes. Each trial consisted of an auditory go cue (synthetic voice vocalizing either *Left* or *Nothing* to indicate the subject the nature of the trial), see Fig. 2. Six seconds after the go cue and auditory stop signal was given by means of the same synthetic voice uttering the word *Stop*. Micro-breaks of six seconds were provided after each trial to give the subject a chance to gaze away from the fixation cross, and for brain electrical patterns to return to baseline levels. Subjects were also instructed to try to minimize movement towards the end of these micro-breaks in order to not create electrical activity in the brain or movement artifacts that would propagate to the next trial. The sequence of left and no task trials was randomized. The interval between consecutive trials was 12 seconds.

#### Data Acquisition and Analysis

EEG was recorded reference free with a Biosemi ActiveTwo amplifier from 32 Ag/AgCl active electrodes placed over the scalp using the 10–20 system. No electro-oculogram was recorded. EEG signals were resampled prior to data processing to 128 Hz. For data analysis, we used common average reference (CAR). This method uses the mean of all electrodes as reference.

In order to increase generalisability of our results to real-world applicability we decided not to visually inspect the data for EEG artifacts prior to data analysis. Therefore the EEG signals were not artifact free.

Prior to calculation of spatial filters, the re-referenced EEG signal was filtered in a 8–30 Hz band. The filter used was a zero-phase forward/backward FIR filter. That specific frequency band was chosen because it contains the alpha and beta frequency bands which have been shown to contain the ERD/ERS phenomena useful for imaginary movement detection.

Data was analyzed by custom Matlab scripts using the open source EEGLAB and BCILAB toolboxes. All anonymized participant’s recorded files containing EEG and pupil diameter signal in XDF format are available in the following repository: https://www.dropbox.com/s/lg71rc72g9bj7dj/userdata2.rar?dl=0.

#### Common Spatial Patterns

Due to the different anatomical properties of cortical folding between people and volume conduction, the areas of maximum discriminatory power between experimental conditions for the ERD/ERS phenomena characteristic of hand movement and motor imagery, are not strictly located beneath electrode positions C3 and C4 situated over the motor cortex.

A successful signal processing technique to minimize error rate in classification of motor imagery is to use the method of Common Spatial Patterns (CSP), which constructs subject-specific spatial filters optimized for discrimination among two experimental conditions present in raw EEG data.

CSP spatially filters raw EEG channels to a smaller number of time-series whose variances are optimized for the discrimination of 2 classes, in this work: left hand motor imaginary and a no task condition. The method is based on the simultaneous diagonalization of 2 covariance matrices. The method was introduced to EEG analysis for detection of abnormal EEG [33] but it has since then been successfully applied to the classification of motor imaginary EEG [15, 17].

For analysis, as explained in [16], the raw EEG data is partitioned into class specific epochs. Each epoch is represented as an *NxT* matrix *E*, where *N* is the number of channels (i.e., number of recording electrodes) and *T* is the number of samples per channel. The normalized spatial covariance of the EEG can be obtained from:

$$\begin{array}{c} C=\frac{EE^{\prime }}{trace(EE^{\prime })} \end{array}$$ (1)

Where ′ denotes the transpose operation and the trace function is the sum of the diagonal elements of *EE*′. For each of the 2 distributions to be separated (i.e., the left motor imagery and the no task condition), the spatial covariance ${\bar{C}}_{d},\in [l,nt]$ (here *nt* stands for the no task condition and *l* for the left hand motor imagery condition), is calculated by averaging over all the trials from each class. The composite spatial covariance is given as:

$$\begin{array}{c} C_{c}={\bar{C}}_{nt}+{\bar{C}}_{l} \end{array}$$ (2)

*C*_*c*_ can be subsequently factorized as $C_{c}=U_{c}\lambda_{c}U_{c}^{\prime }$, where *U*_*c*_ is the eigenvectors matrix and *λ*_*c*_ is the diagonal matrix of eigenvalues. From now on, we will assume that the eigenvalues are sorted in descending order.

The variance in the space spanned by *U*_*c*_ can be equalized by applying a whitening transformation.

$$\begin{array}{c} P=\sqrt{\lambda_{c}^{-1}}U_{c}^{\prime } \end{array}$$ (3)

This achieves that the eigenvalues of *PC*_*c*_ *P*′ are made equal to 1. If ${\bar{C}}_{nt}$ and ${\bar{C}}_{l}$ are transformed as:

$$\begin{array}{c} S_{nt}=P{\bar{C}}_{nt}P^{\prime } \end{array}$$ (4)

and

$$\begin{array}{c} S_{l}=P{\bar{C}}_{l}P^{\prime } \end{array}$$ (5)

then *S*_*nt*_ and *S*_*l*_ share common eigenvectors:

$$\begin{array}{c} \text{If}\;\;\;S_{nt}=B\lambda_{nt}B^{\prime }\;\;\text{then}\;\;\;S_{l}=B\lambda_{l}B^{\prime }\;\;\text{and}\;\;\;\lambda_{nt}+\lambda_{l}=I \end{array}$$ (6)

Where I is the identity matrix. Because, the sum of two corresponding eigenvalues is always one, the eigenvector with the largest eigenvalue for ${\bar{S}}_{nt}$ has the smallest eigenvalue for ${\bar{S}}_{l}$ and vice-versa. Hence, the eigenvectors *B* can be used for the classification of the two distributions corresponding to left motor imagery and the no task condition. The projection of the whitened EEG onto the 1^*st*^ and the last eigenvectors in *B* (i.e., the eigenvectors corresponding to the largest *λ*_*nt*_ and *λ*_*l*_) will generate feature vectors optimized for discriminating 2 populations of EEG in the least squares sense [16].

Using the projection matrix *W* = (*B*′ *P*)′, the mapping (decomposition) of a single trial *E* is produced by:

$$\begin{array}{c} Z=WE \end{array}$$ (7)

**Fig 3:**
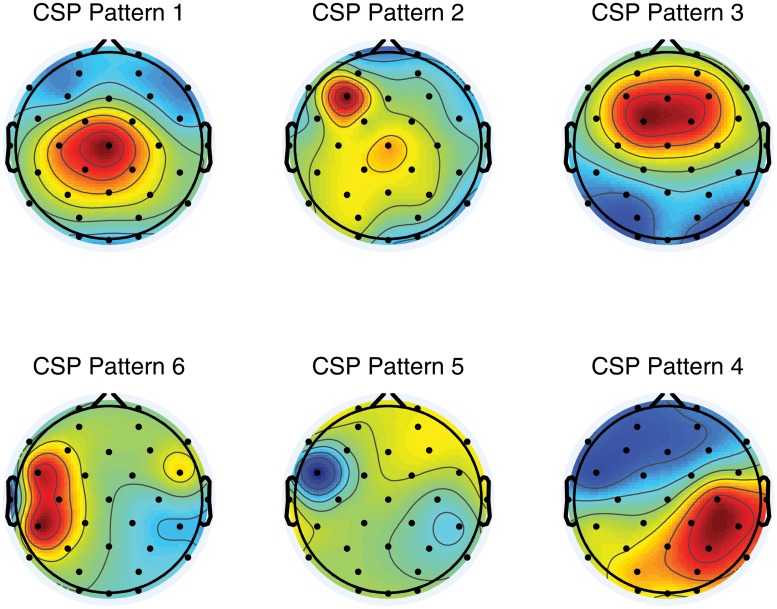
Common Spatial Pattern Maps. The Figure illustrates a set of common spatial patterns (CSPs) filters of a single participant in the study. The CSPs are optimized for the discrimination of left hand motor imagery from a control rest condition.

The columns in *W* ^−1^ are the CSPs, i.e., the time invariant EEG source distribution vectors. See Fig. 3 for an illustration of a 3-pair set of CSPs scalp projections generated for one participant in the user study.

### Classification

The raw EEG signal is filtered by Equation 7 in order to generate the features used for classification. For each of the classes (no task condition and imaginary left hand grasping motion condition), the variance of only a reduced number *m* of the signals are suitable for discrimination and they are the ones used for the construction of the classifier. In our study, the CSP feature vector had a length of 6, since 3 pairs of CSPs were computed for each experimental block. The signals that maximize the difference of variance of left motor imaginary versus the no task condition are the ones that are associated with the largest eigenvalues *λ*_*nt*_ and *λ*_*l*_. The derived feature vectors of the *Left* and the *No task* trials are used to estimate a linear discriminant analysis (LDA) classifier.

For deriving the feature based on pupil diameter, we averaged the diameter of the right and left pupil diameter. We have tried out classification for detection of motor imagery using a range of different pupil diameter derived signal statistics during the trial: mean, derivative, maximum, minimum, range, variance, skewness and kurtosis, see Table 1. The Table shows the average value of the statistics for both classification conditions as well as the threshold used for classification of a trial using the corresponding metric. Finally the last column of Table 1 displays the classification accuracy obtained for the different statistics. We performed an exhaustive combination of all features. We found that only the trial mean was discriminative enough as a feature for classification, with the other features not adding any additional value for classifying between the *Imaginary Movement* and the *No Task* conditions. We also tried to add additional dimensions to the classification vector by using the pupil diameter averages between time intervals [3 4], [4 5] and [5 6] within a trial but we failed to see any discernible effect with respect to just adding the average pupil diameter over the entire epoch

**Table 1:** Different Metrics of Pupil Diameter. We investigated several pupil diameter derived metrics to try to find the feature that obtains better classification accuracy. The average value for the statistics for all participants in the user study for both experimental conditions was calculated. The middle point between the previous two values was used as a threshold for classification. The classification accuracy is shown in the rightest column with the standard deviation. In the last row, FBA stands for mean Frequency Band Amplitude.

Pupil Diameter Statistics	Average value for Motor Imagery	Average value for Control Condition	Threshold Employed For Classification	Classification Accuracy
Mean	0.131	-0.030	0.050	73.3 ± 8.1
Derivative	0.161	-0.023	0.069	66.1 ± 10.2
Max	0.376	0.227	0.302	65.1 ± 12.5
Min	-0.181	-0.353	-0.267	64.9 ± 11.4
Range	0.587	0.361	0.471	51.7 ± 15.1
Variance	0.026	0.021	0.023	51.9 ± 15.2
Skewness	-0.375	-0.276	-0.326	47.9 ± 8.7
Kurtosis	3.927	4.503	4.215	51.7 ± 9.1
0–0.2 Hz FBA	0.025	-0.006	0.009	70.0 ± 8.9

The pupil diameter feature was derived by baselining the trial epoch with data from -2000ms before the experimental go cue (Left or Nothing) to the instant of the go cue (0ms mark in the trial). The average baselined pupil diameter from 500ms after the go cue until 6000ms was calculated and that number was the pupil derived feature used for classification.

For classification using just the pupil data, the normalized mean pupil diameter over the trial was calculated. If this number was above 0.05, see Table 1, the trial was considered to belong to the motor imaginary condition otherwise it was deemed to belong to the control condition.

We carried out a frequency domain analysis of the pupil signal over the entire 5 minutes of data gathering in each block. However, the pupil signal is non-stationary, i.e. its spectrum is varying in time, while the popular fast Fourier transform requires a stationary signal. Hence, a short-time Fourier transform was used within each trial for analysis of the signal in the frequency domain. The Fourier coefficients were divided by the number of time points in the trial to scale the coefficients to the amplitude of the original data. We could not analyse frequency content above 15Hz given the sampling rate of the eye tracker used was 30Hz.

In this work we test the performance of a traditional BCI using only CSP derived EEG features in classification against the performance of a classifier that uses EEG features plus the pupil diameter feature. For completeness, we also report the performance of classification using just the pupil diameter feature alone. For combining the EEG features with the pupil diameter feature we simply increase the dimensions of the vector with EEG-derived features fed to the LDA classifier by 1, to introduce the feature normalized average pupil diameter during the trial active period.

**Fig 4:**
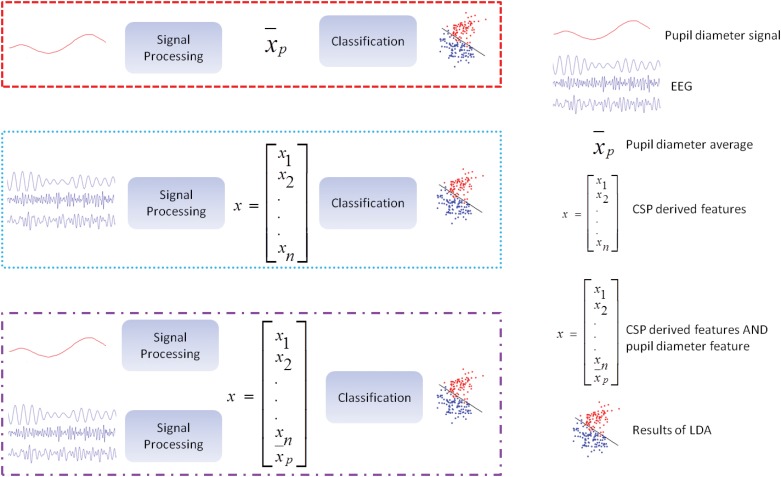
Illustration of the Different Classification Approaches. A legend of the symbols used in this figure is contained in the right side of the figure. The dashed red box encapsulates the method used to classify motor imagery using only the pupil signal. A moving average of the pupil diameter is continuously updated and compared to a reference baseline period right before trial start. The dotted blue box encapsulated the method used to classify motor imagery using the EEG derived signals and the CSP algorithm. The alternately dashed and dotted purple box illustrates our proposed method by which the average pupil diameter is added as an extra feature to the EEG derived feature vector used for classification. After extension of this vector, classification is carried out.

Fig. 4 illustrates the different approaches used for classification. All methods consisted of a signal processing part that filter the raw signals and extracted features from them and a classification part using different combinations of features that employs LDA. In Fig. 4, within the dashed red box, the method that used just pupil diameter for classification is depicted. The dotted blue box encompasses the methods using only EEG derived features from the CSP spatial filter. The alternatively dashed and dotted purple box illustrates the EEG derived feature vector with the addition of the pupil diameter feature before classification.

Two types of training approaches were tested. In one, each block of experimental data was evaluated using 10-fold cross validation within the block and the average error of both blocks was reported. In the alternative approach, a block of data was used in its entirety for training and the remaining block was used for testing from which to derive the error rate.

### Performance Measurements

We monitored the performance of the paradigms being tested using the following metrics:

$$\begin{array}{c} \text{P}=\frac{\sum \text{True}\;\text{Positives}+\sum \text{True}\;\text{Negatives}}{\text{N}} \end{array}$$ (8)

$$\begin{array}{c} B=\log_{2}N+A\log_{2}P+\left(1-P\right)log_{2}\frac{1-P}{N-1} \end{array}$$ (9)

$$\begin{array}{c} \kappa =\frac{P-P_{0}}{1-P_{0}} \end{array}$$ (10)

where *N* is the number of targets, *P* is the accuracy of the classifier, *B* is the number of bits transmitted per trial, *P*_0_ is the probability of guessing the correct class due to chance and *κ* is the kappa coefficient.

### Correlation and Mutual Information

To measure the association between the predictive power of the pupil signal and the EEG signal we have carried out a Pearson correlation analysis to rate the strength of statistical dependence between both variables. Pearson correlation provides an index of linear statistical dependence. But since non-linear dependencies can elude Pearson correlation, we have also estimated the mutual information between the predictive power of the pupil signal and the EEG signal. The mutual information (MI) between two random variables is a measure of the variables’ mutual dependence. MI is more general than the correlation coefficient and determines how similar the joint distribution is to the products of factored marginal distribution. Formally, the mutual information of two discrete random variables X and Y can be defined as:

$$\begin{array}{c} I\left(X;Y\right)=\sum_{y\in Y}\sum_{x\in X}p\left(x,y\right)log\left(\frac{p(x,y)}{p(x)p(y)}\right) \end{array}$$ (11)

where *p*(*x*,*y*) is the joint probability distribution function of *X* and *Y*, and *p*(*x*) and *p*(*y*) are the marginal probability distribution functions of *X* and *Y* respectively.

### Participants

We recruited 30 participants aged from 15 to 61 years, with and average of 38 (*SD* = 9.69). Genders were split equally with half of the participants female and half male. All but three participants were right handed and all had normal or corrected to normal vision. The volunteers were compensated with a 30AUD voucher for their participation.

## Results

Table 2 shows the results of the different classification methods and classifier training approaches and the metrics used to evaluate performance: accuracy, Kappa coefficient and Information Transfer Rate.

**Table 2:** Experimental Results. Average classification accuracy, Standard deviation, Kappa Coefficient and Information Transfer Rate (ITR) in bits/trial and bits/min for all classification methods split by the training and test blocks experimental approach and the 10-fold cross-validation within block experimental training approach.

		separate training/test blocks		cross-validation within block	
	Pupillometry	EEG	EEG+pupil	EEG	EEG+pupil
Kappa Coefficient	-0.47	-0.34	-0.57	-0.51	-0.67
ITR (bits/trial)	0.16	0.08	0.25	0.20	0.35
ITR(bits/min)	1.64	0.85	2.52	1.99	3.49

Given that the pupil diameter provided the best classification accuracy of all the pupil metrics investigated, see Table 1, we used this feature in the multi-modal classification approach using EEG derived features and the pupil diameter average within a trial.

**Fig 5:**
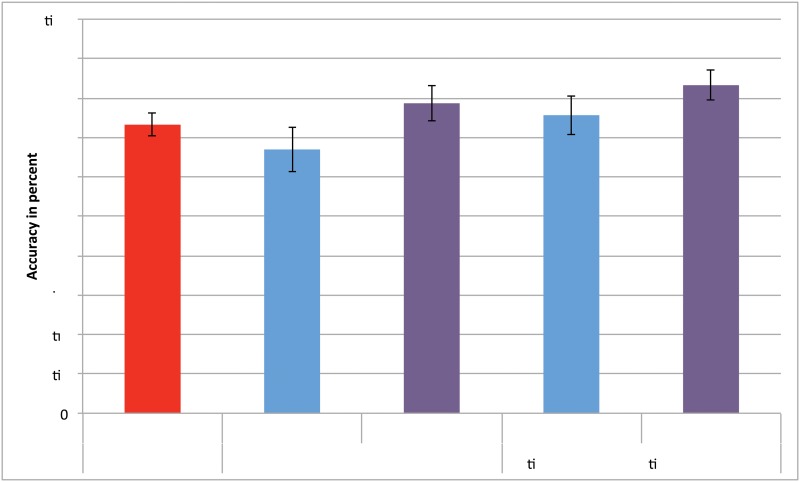
Classification accuracy. Average classification accuracy for the different methods; error bars show Confidence Interval. The figure is color-coded to match the bounding boxes in Fig. 4 that identify the different paradigms employed in data analysis.

We compared the classification accuracy for motor imagery detection between using the pupil diameter feature, the EEG derived features and the combination of the pupil diameter feature with the EEG derived features consisting of a simple addition of the average pupil diameter over the epoch to the EEG derived feature vector. We performed this comparison for a training approach using 10-fold cross-validation within each block of gathered data and an alternative training approach using a block of data for training and the remaining block for testing (Fig. 5).

With the training-block and test-block approach a repeated measures ANOVA on the accuracy metric revealed a significant difference between these methods (*F*(1.47,42.56) = 9.34, *p* <.01, $eta_{\text{p}}^{2}=.24$) (df have been adjusted using Greenhouse-Geisser correction where necessary). Post-hoc multiple comparisons (with Bonferroni correction) showed that classification accuracy was significantly higher with EEG+pupillometry than with EEG alone.

With the 10-fold cross-validation we also found a significant difference between the classification methods for the accuracy metric (*F*(1.43,41.51) = 11.47, *p* <.01, $eta_{\text{p}}^{2}=.28$). Classification accuracy was significantly higher with EEG+pupillometry than with either EEG or pupillometry alone. Classification accuracy did not differ significantly between EEG and pupillometry.

To see how combining EEG with pupillometry may improve classification accuracy, especially for BCI illiterate users, we compared EEG and EEG+pupillometry classification accuracy separately for those with low EEG classification accuracy and those with higher classification accuracy on the EEG data. As potentially BCI illiterate participants we took those falling into the lower quartile. The lower quartile was chosen based on an estimated BCI illiteracy incidence rate of 20–30% [22] (in our case participants with EEG classification accuracy of 56% or lower. A paired t-test for this subgroup showed a significant difference (*t*(8) = −5.09, *p* <.01) between EEG and EEG+pupillometry with a very large effect size (*d* = −1.89). Mean EEG accuracy for this group was at chance level with *M* = 50% and *SD* = 5% compared to a mean EEG+pupillometry accuracy of *M* = 69%, *SD* = 12%). The sub-group with mean EEG classification accuracy greater than the first quartile had a mean EEG accuracy of *M* = 74%, *SD* = 13% and EEG+pupillometry accuracy of *M* = 83%, *SD* = 11%, which also was significantly different (*t*(20) = −4.11, *p* <.01) with still high, though smaller effect size (*d* = −1.07).

**Fig 6:**
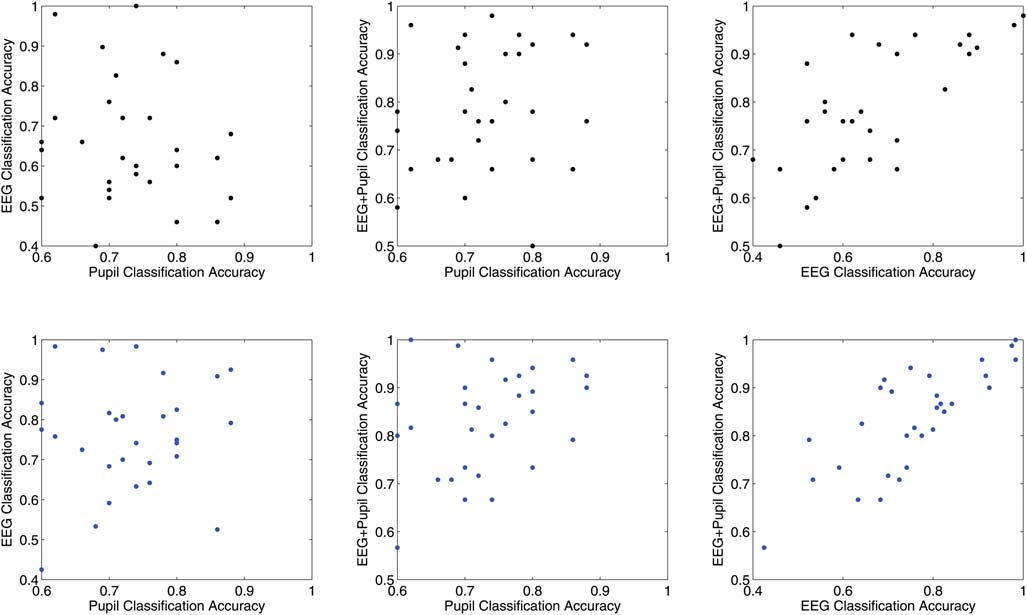
Scatter Plots of Correlation Analysis Between Different Classification Feature Sets. The Figure illustrates in the top row with black dots the classification accuracy with the separate training and test set approach and in the bottom row with blue dots the classification accuracy with the cross validation training approach.

In the training-block test-block approach and the cross validation training approach, the classification accuracy using just pupil diameter showed a very small correlations with the EEG and the EEG+pupillometry based classification (see Table 3). The correlation between EEG based classification and classification with EEG+pupil diameter was *r* = .72, (*p* <.01) for the separate training and test blocks and *r* = .78, (*p* <.01) for the cross-validation approach. For illustration purposes, Fig. 6 shows the scatter plots of comparing the different classification accuracies of different feature sets for the separate training and test blocks (in black, top row) and the cross validation approach (in blue, bottom row).

**Table 3:** Correlations. Correlations of classification accuracy between pupil diameter and EEG, pupil and EEG+pupil as well as EEG+pupil and EEG; The table is split by the training and test blocks experimental approach and the 10-fold cross-validation within block experimental approach.

	separate training/test blocks	cross-validation within block
pupil * EEG	-0.12 (*p* = .51)	0.13 (*p* = .49)
pupil * EEG+pupil	0.18 (*p* = .35)	0.33 (*p* = .08)
EEG * (EEG+pupil)	0.72 (*p* <.01)	0.78 (*p* <.01)

We have also calculated the mutual information between the pupil time series predictive power and the eeg signal to be 1.17 bits (*p* < 0.01) in standard-Z values by performing permutation test. The entropy for the pupil, eeg and joint signals was 3.1609, 3.0554, 5.0468 respectively. The number of bins used for discretization based on Freedman-Diaconis rule was 10.

**Fig 7:**
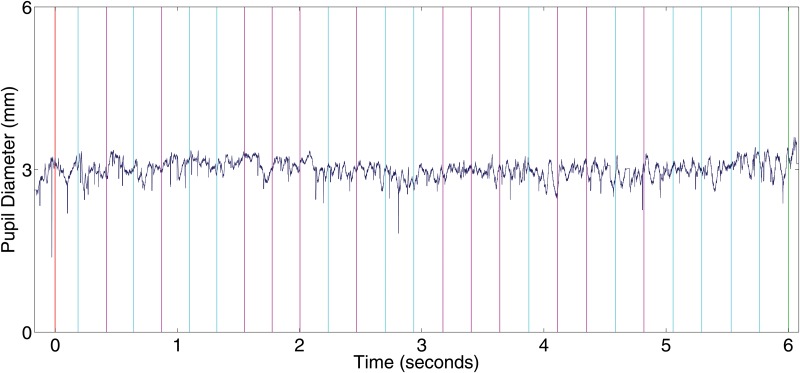
Pupillary Diameter Time Course for a Single User During an Entire 5 Minutes Block. The Figure illustrates the inherent noise of the signal and the difficulty to discern patterns of motor imagery by pupil dilation with the naked eye. Trial classes (left hand imaginary movement and nothing condition) are marked in the Figure as pink and cyan vertical bars respectively. The beginning of the experiment is marked with a red vertical line. The end of the experiment is marked with a vertical green line. Notice that the second half of the interval between vertical bars corresponds to the micro-break given after the auditory stop cue within each trial and hence it was not used in the calculation of the average pupil diameter.

**Fig 8:**
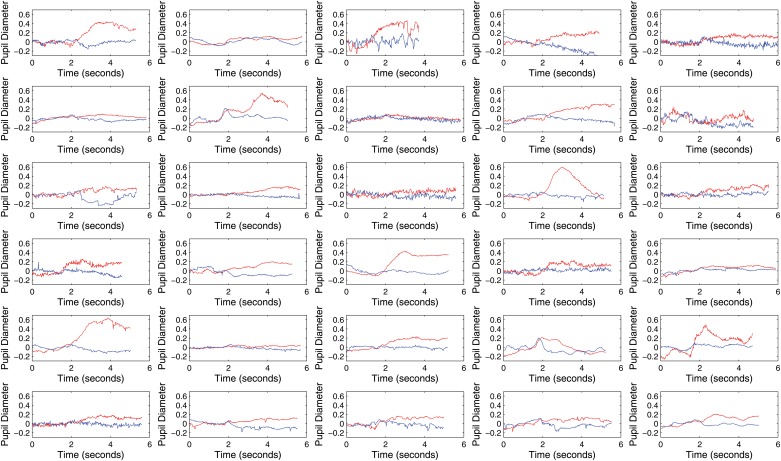
Pupil Diameter Time Course Averages within Experimental Epochs for All Participants. The figure illustrates the average time course of pupil diameter within all trials for both experimental conditions. The red color denotes the left hand motor imagery class and the blue line the no task condition. Pupillary diameter enlarges during the the motor imagery condition epochs and its average is higher than the average pupil diameter during the control condition for almost all participants.

Fig. 7 shows a time series of the pupil diameter signal for an entire 5 minute data gathering block for one study participant to illustrate the appearance of the pupil diameter signal over an entire block. Fig. 8 presents the average pupil diameter of all trials in a block for all participants in the user study. The blue curve represents the control (rest) condition and the red curve corresponds to the active (motor imagery) condition for all the participants in the experiment. Notice that the pupil diameter variable has been normalized using as a baseline the time period of 2000ms prior to the trial go cue (first 60 gaze samples in the Figure). The enlargement of the pupil in the trials involving motor imagery in contrast to the control condition is apparent for almost all participants. The effect however differs in size among different subjects.

**Fig 9:**
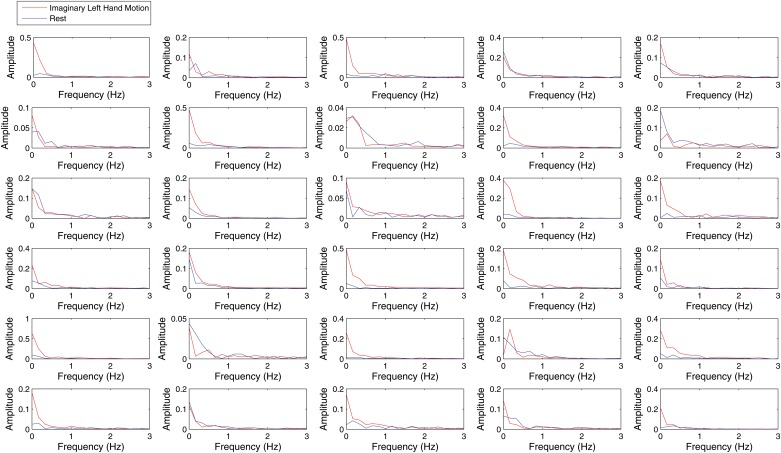
Pupil Diameter Frequency Response within Experimental Epochs for All Participants. The figure illustrates the average frequency domain response of pupil diameter within all trials for both experimental conditions for all participants. The red color denotes the left hand motor imagery class and the blue line the no task condition. Pupillary oscillations in the frequency band between 0 and 0.3Hz seem larger in the left motor imagery trials during the motor imagery condition in most but not all participants.

Fig. 9 shows the frequency domain response of the pupil diameter signal within trials. We did not find any differences in the frequency content between both experimental conditions above 1Hz. In the frequency band between 0 and 0.3Hz, there was a consistent higher amplitude of the signal in the frequency domain for the motor imagery condition in comparison to the control condition for most but not all participants. When the mean amplitude of the frequency domain representation in the 0–0.3Hz band is used as a feature for classification of motor imagery, the performance is similar to the classification accuracy obtained by the average pupil diameter over the entire trial in the time domain, see Table 1. The Pearson correlation coefficient of both variables was 0.75.

**Fig 10:**
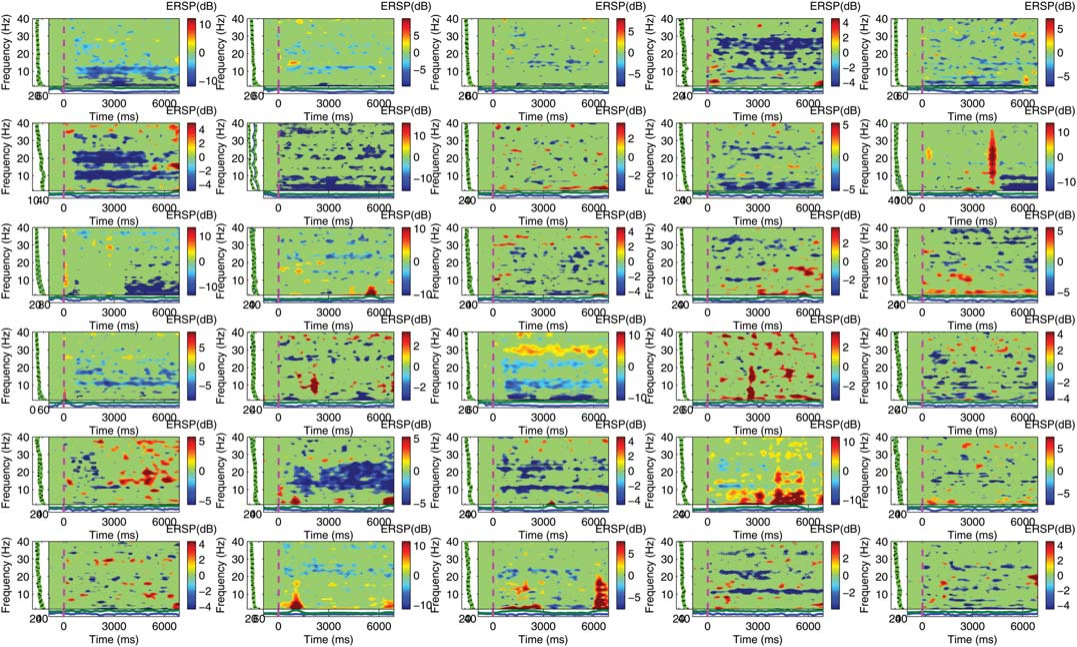
Event Related Spectral Perturbation for all Participants During Left Hand Motor Imagery. The figure illustrates the frequency spectrum time course of EEG activity within motor imagery epochs for each participant. Event Related Desychronization in *α* and/or *β* frequency bands is apparent in some but not all participants. This is to be expected in a large pool of subjects due to the anatomical differences of cortical foldi and the resulting effects of volume conduction.

The power spectral perturbation of all participants for left hand motor imagery in electrode C4 are illustrated in Fig. 10. Notice that the decrease of power in the alpha band for the imagery condition with respect to the control condition is not obvious in all participants. This can be explained due the different structure of cortical foldi among different subjects and volume conduction which makes it possible that an ERD event corresponding to left hand motor imagery might not be present at electrode C4 but still be apparent elsewhere.

## Discussion

The results of this work show the limitations of EEG-based motor imagery detection using mu rhythms and the advantages of a multi-modal approach that combines the pupil diameter feature with the EEG derived features to improves the performance of the classification. Our results show that there was no statistically significant difference between motor imagery classification using EEG features and classification using just the pupil diameter feature. This is quite remarkable since the pupil feature is just 1 dimensional in comparison with the EEG which contains 31 dimensions. Particularly relevant for applying our approach to real world scenarios is that monitoring the pupil size is much less cumbersome for both the subject and the investigator than monitoring EEG. An additional advantage of the pupillometry approach is that it does not require training data at all since it uses a fixed threshold to discriminate between the classes and a continuously updated normalization approach (the baseline period prior to the trial go cue). Furthermore, the pupil signal does not need manual data cleansing as the EEG signal often does. However, the pupil signal is very sensitive to environmental brightness dynamics. Hence, we do think of both types of signals, EEG and pupillometry, as complementary to each other in our proposed multi-modal system. As shown in our results, combining both signals outperforms classification accuracy from either signal in isolation

There are, however, some limitations with our proposed approach. We attempted to distinguish between just two relatively easily separable classes: left-hand motor imagery and a no task condition which presumably is distinct enough from active motor imagery. State of the art BCIs are able to discriminate up to 4 degrees of freedom using motor imagery from the right and left hand, feet and tongue. Our goal here, however, was not to stretch the boundaries of BCI’s accuracy for a maximum number of degrees of freedom, but rather to show the potential of our multi-modal approach to improve the detection of motor imagery for using it as a switch-like interaction technique.

The pupil diameter obviously not only reacts to cognitive processes, the pupil is also sensitive to changes in ambient light and stimulus brightness. Pupil changes due to such variables could act as confounding factors limiting the external validity of our approach. Ambient light changes, however, should not invalidate pupillometry as a means of assessing motor imagery, since changes in pupil diameter are normalized continuously to a baseline period immediately prior to the event signaling the nature of the trial, (*Left* or *Nothing*), as we did in this work. Hence, slow wave changes in ambient light could be accommodated and should not limit the technology’s applicability. The brightness of the stimulus at which the subject is gazing at is more problematic since the stimulus brightness variability can affect pupil diameter. In order to control for such potential confound, we controlled stimulus brightness by making subjects fixate on a cross in the center of a gray screen and presenting the trial condition by sound cues. This is a common experimental setup for motor imagery based BCI experiments in order to avoid eye movement artifacts. But we acknowledge that such constraints may limit the external validity of the results. Nonetheless, mechanisms that take pupil diameter changes due to stimulus brightness into account can be envisioned. Such an approach could be conceptualized as a software system that monitors the point of regard of user’s gaze on the screen and evaluates the screen brightness around the user’s gaze in order to discount the brightness of the area gazed at from pupil diameter changes. That way, pupil changes in diameter could be invariant to changes in stimulus brightness. In this work we made a first step to provide evidence that pupil diameter time series contain information about a subject’s motor intent. While we still face a few practical challenges to take full advantage of this information channel, we demonstrated the potential of the pupil diameter signal in adding useful information to the detection of motor imagery.

Other limitations are mainly linked to our chosen study paradigm. We designed our study with the intention of increasing the generalisability of our findings in terms of maximizing the practicality of systems incorporating motor imagery detection. Under ideal conditions such as fully controlled lab environments, manual curation of data or exclusion of certain subjects, EEG based BCI may work reasonably well. However, such conditions are often far from practical for regular use of such technologies. And it is under such non-ideal conditions in which our proposed multi-modal approach has the greatest potential benefit.

The EEG-based motor imagery detection accuracy achieved in this work is not as high as what has been reported by state of the art BCI systems. However, we generated the raw EEG data under rather loose conditions. We only used 32 EEG channels which limited our ability to employ high spatial frequency filtering methods such as the Laplacian filter to improve the spatial resolution of our data. Instead, our approach reduced setup times and inconvenience to the study participants. Furthermore, subjects underwent very short sessions of only 5 minutes for each of the two data gathering blocks, which is substantially shorter than data gathering sessions reported in other studies, which often exceed 15 minutes. We had two reasons for using a relatively short training session: to minimize fatigue of study volunteers, and to highlight one of the advantages of our multi-modal approach to motor imagery detection. The improvement in accuracy that the multi-modal approach affords reduces the amount of training necessary to calibrate the system and hence, it makes it easier on human subjects to use the technology.

We chose not to manually curate the recorded raw EEG data which is often done in EEG data analysis to filter out artifacts. All trials went into the training and test stages independently of oculomotor activity or other EEG artifacts in the data. Again, in EEG studies the data is often manually curated by filtering out trials in the calibration that contain artifacts.

There was no significant correlation between the classification accuracy of the EEG and the pupil feature alone, see Table 3. Correlation was larger but still not significant between the results of classification using the pupil feature and using the EEG and pupil diameter. The only significant correlation was between the EEG classification and the EEG+pupil classification. The weak correlation between the performance of the classifier using just the pupil and the performance of the classifier using just the EEG signal indicates the potential of both signals to the complementary to one another. This is corroborated by the analysis of a subset of participants with particularly low performance on EEG-based classification. For these participants the improvement in performance when using the pupil diameter was particularly stark. Hence, motor imagery illiterate users might particularly benefit from the pupil diameter feature since both signals seem to only weakly correlate

BCI illiteracy refers to the inability of a subset of people to use certain BCI paradigms. BCI illiteracy is a well described phenomenon (e.g. [22]). In the case of motor imagery based BCIs, illiteracy is thought to affect between 20–30% of potential users. In our experiments, we chose participants randomly and we did not filter out anyone with poor motor imagery classification performance. Hence, our EEG dependent accuracy results may be biased by the inclusion of several subjects irrespective of them potentially being BCI illiterate for the mu rhythm dependent motor imagery detection paradigm. The benefits of our approach are particularly beneficial for the subset of potential BCI users affected by motor imagery illiteracy. As shown in the Results section, overall classification accuracy markedly improves for such a subset of users when the BCI is extended with the pupil diameter signal.

We found similar trends in the data for both training approaches (within block cross validation and separate training and test blocks) in the sense that the EEG plus pupillometry modality approach obtained better classification accuracy than the modality using only EEG derived features. Accuracy performance was generally higher using the within-block cross-validation training approach. This is due to the presence of non-linearities in EEG data that often markedly emerge between data gathering blocks. EEG features change over time due to changes in electrode/scalp impedance, subject fatigue or electrode positioning. Hence the extrapolation of models from one block to another suffers from a decrease in performance in comparison to training and testing within the same block using cross validation.

The results obtained for motor imagery detection using only the pupil diameter feature is rather similar to classification performance using just EEG derived features. While the difference between EEG+pupillometry and pupillometry alone was significantly better for the multi-modal paradigm in the cross-validation approach, it was superior but not statistically significant with the training-block test-block approach. The difference in accuracy was approximately 5 percentual points. This is quite remarkable since just using the pupil feature to detect motor imagery intention is much less invasive and inconvenient for the subject than using EEG (which involves attaching 32 wet electrodes to the scalp). However, as discussed above, the pupil signal also suffers from several limitations. Hence, both psycho-physiological signals can be thought of as complementary. At this stage it is reasonable to assume that the pupil channel can only be used for binary motor imagery detection in stark contrast with EEG based BCIs that can distinguish up to 4 classes of motor imagery albeit with reduced accuracy than when just distinguishing one class of motor imagery from a rest condition. Additionally, the rather short training and test blocks used in our experiments limit the potential of EEG based classification to exceed the pupil diameter classification performance.

An implicit assumption of our system is that users are in an idle state whenever they do not want to use the BCI. In realistic application scenarios users might engage in specific mental activity which could trigger pupil enlargement while they do not want to activate the mental switch. However, it is unlikely that such an event might also trigger simultaneously the same sort of desynchronization observed in the EEG signal during motor imagery. On the other hand, certain types of motor movement might trigger similar patterns to the ones detected in left hand motor imagery, but they are unlikely to cause pupil enlargement. Therefore, we did not specifically study the ability of our system to discriminate between mental imagery and specific mental tasks other than the rest condition of our experiments. However, the performance of our system when users engage in other sorts of mental activity such as for instance mental arithmetic or stressful situations could be an interesting venue for further exploration.

## Conclusion

EEG-based BCIs offer direct communication channels between humans and computers which afford novel interaction methods for individuals with high degrees of motor impairment. Although considerable progress has been made over the last decades to improve the accuracy of EEG-based BCIs, performance remains sub-optimal when compared to mechanical interaction devices such as mice or switches. Often, the BCIs only operate under very constrained conditions such as one or several of the following: laboratory environments, controlled light conditions, synchronous paradigms, constrained gaze and muscle activity, manually curated data, expensive hardware equipment, high number of electrodes, gel based electrodes and long training sessions. All these limitations make BCIs very inconvenient for the average user and limit the practical application of this technology.

Improvements in BCIs accuracy over time seem to be following an asymptotic trajectory, suggesting the technology might be approaching its physical limitation in terms of accuracy [22]. In this context, we argue that the development of multi-modal approaches that combine several electro-physiological signals to detect intention for switch-like functionality is a viable approach to improve the accuracy and to maximize the information transfer rate between the human and the machine. Second order dilations of the pupil are related to cognitive processes and provide an index for a diverse set of mental and physiological events. The pupil generates a strong response to cognitive tasks such as cognitive load or the utilization of working memory. Based on research suggesting that motor imagery may cause fatigue and our own observational evidence with subjects reporting that engaging in motor imagery requires mental effort, we speculated that motor imagery would trigger a pupil response that could be detected with a modern video-based oculography system. We have been able to show that this is the case and that monitoring EEG signals and pupil diameter signals simultaneously can improve the detection accuracy of motor imagery. We did this by adding the pupil diameter as an additional feature in a classifier that uses traditional features extracted from an EEG signal. While the improvement in accuracy with respect to EEG-based BCIs might be perceived as just incremental, its statistical significance highlights that the proposed multi-modal approach represents a viable approach for the creation of more robust and reliable BCIs and hence holds considerable promise for further exploration.
