# Correction: Improving the Performance of an EEG-Based Motor Imagery Brain Computer Interface Using Task Evoked Changes in Pupil Diameter

**DOI:** 10.1371/journal.pone.0133095

**Published:** 2015-07-14

**Authors:** The PLOS ONE Staff

There are errors in Fig 5. Please see the correct Fig 5 here. The publisher apologizes for these errors.

**Fig 5:**
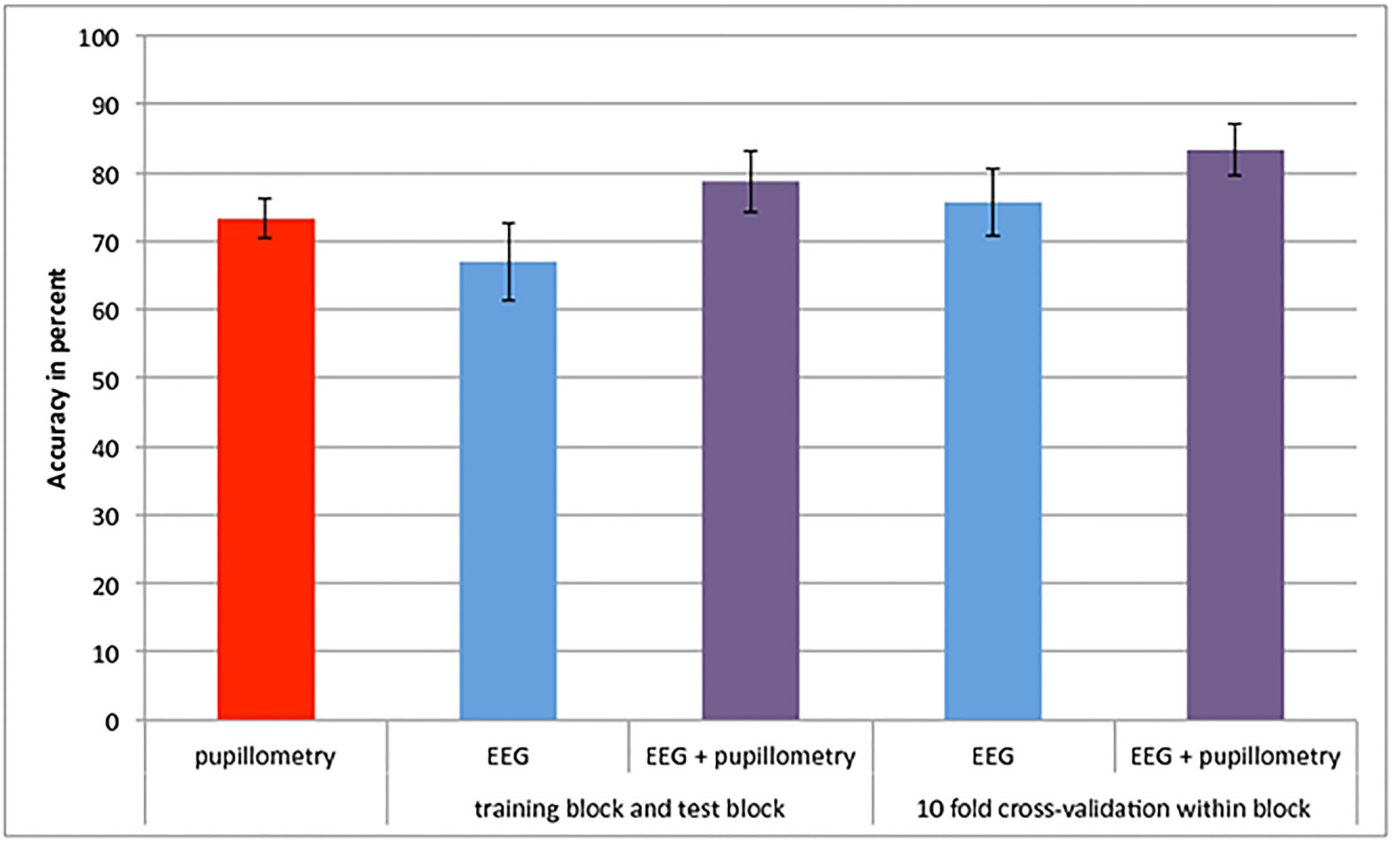
Classification accuracy. Average classification accuracy for the different methods; error bars show Confidence Interval. The figure is color-coded to match the bounding boxes in Fig. 4 that identify the different paradigms employed in data analysis.
